# Implications of COVID-19: The Effect of Working From Home on Financial and Mental Well-Being in the UK

**DOI:** 10.34172/ijhpm.2021.33

**Published:** 2021-04-21

**Authors:** Eleftherios Giovanis, Oznur Ozdamar

**Affiliations:** ^1^Department of Public Finance, Nazilli Faculty of Economics and Administrative Sciences, Aydın Adnan Menderes University, Aydın, Turkey.; ^2^Department of Economics, Faculty of Economics and Administrative Sciences, Izmir University Bakırcay, Izmir, Turkey.

**Keywords:** COVID-19, Difference-in-Differences, Financial Well-Being, Mental Well-Being, Working From Home, United Kingdom

## Abstract

In response to the threat posed by coronavirus disease 2019 (COVID-19), the UK prime minister announced on the 23rd of March strict lockdowns and introduced a new way of living and working, at least temporarily. This included working from home (WHF) wherever possible. Many experts from the IT industry were long arguing about the potential for WFH, which suddenly now became indisputable. The objective of this study is to evaluate the impact of WFH on the individuals’ perception about their future financial situation and their mental well-being. We apply a difference-in-differences (DiD) framework using data from the UK Household Longitudinal Study (UKHLS) combined with the UKHLS COVID-19 survey conducted in April 2020. Our findings suggest that those who have not experienced a shift from working at the employer’s premises to WFH became more concerned about their future financial situation. However, we find that WFH has a negative impact on mental well-being. On the other hand, we find no difference in the mental well-being when we consider those who work from home on occasion. The findings of this study have policy implications for government, firms and health practitioners. In particular, a balance between WFH and at the employer’s premises may provide both financial security and maintain the mental and psychological well-being at satisfying levels.

## Introduction

 The novel coronavirus disease 2019 (COVID-19), has posed new challenges to the society, prompting people reconsider a wide variety of practices, from work, to daily tasks, to basic travel and recreational activities. Not only has this had an individual effect, but it has also had an economic impact on countries as a whole, bringing a variety of economic sectors to a complete halt. Since the outbreak of the novel coronavirus, countries have taken numerous steps to avoid its spread. These initiatives have however had an enormous impact on the world economy, particularly in countries that were hard hit by the coronavirus, such as Italy, Spain, the United States and the United Kingdom. The UK government, in response to the COVID-19 outbreak and spread, shut down almost every school, companies, social venues, and banned all “non-essential” travel outside the home. A prolonged lockdown, however, is expected to severely harm the UK economy, leading to sharp increases in unemployment and deterioration in financial and mental well-being.^5^ In a recent paper, Davillas and Jones used data from wave 9 of the UK Household Longitudinal Study (UKHLS) and the UKHLS COVID-19 survey in April 2020 to explore the impact of lockdowns in the United Kingdom.^1^ Their findings suggest a significant and systematic drop in the 12-item General Health Questionnaire (GHQ-12) (which measures people’s overall psychological well-being) from 18.3% before the lockdown period to 28.3% during the lockdown.

 To alleviate the negative impact of unemployment, governments and firms across the United Kingdom and the world have implemented welfare programmes and flexible employment schemes. The main objective of this study is to explore the impact of working from home (WFH) on the subjective financial and mental well-being of workers in the United Kingdom.

 Though the number of people *WFH* on a full-time or part-time basis has been gradually increasing over the last several years,^2^ the pandemic has undoubtedly fast-tracked the adoption of *WFH*. Prior to the pandemic, debates about the future of work-life balance were hazy and often questioned. COVID-19 forced people to make a choice, and with the environment requiring rapid adaptation, many companies opted to try WFH. In a scenario such as the COVID-19 pandemic, *WFH* has proven itself an important aspect of ensuring business continuity, while under normal circumstances its benefits include reduced commuting time and increased opportunities for employees to concentrate on their work tasks.

 However, risks can also occur, such as longer working hours, feelings of isolation and loneliness, especially for individuals living alone, and lack of contact with fellow employees.^3-5^ Moreover, in such an urgent and unexpected situation as the COVID-19 pandemic, workers may be unprepared physically and mentally to meet the challenges posed from *WFH*. Although there is empirical evidence about the impact of flexible employment schemes on well-being,^6,7^ little is known about its impact on the financial and mental well-being of individuals who have experienced a sudden shift from the employer’s premises to WFH. In particular, earlier studies have explored the impact of *WFH* on financial and mental well-being, but the shift from working at the employer’s premises to home was expected and planned.Previous studies show that *WFH* can have a positive impact on the employee loyalty to the organization, productivity, job and financial satisfaction.^8-10^ Furthermore, *WFH* can increase time available for other activities; however, this “extra” time is not always spent on leisure activities, but is often filled with other paid work or household chores.^11,12^ Commuting to work can lead to a series of adverse outcomes including health problems and increased stress.^13,14^ Furthermore, teleworking may be particularly advantageous for female workers, as women continue to carry out the majority of household responsibilities in developed countries and teleworking may allow women to better manage their work and household responsibilities.^15,16^

 On the other hand, the concluding remarks about the impact of *WFH* on mental health and well-being are mixed. For instance, Giménez-Nadal et al, using the well-being module from the American Time Use Survey for the years 2012 and 2013, found that male commuters experience higher levels of negative feelings while working than do teleworkers.^17^ While research shows that *WFH* can reduce stress from commuting, it is also associated with feelings of isolation and mental distress due to long working hours and overtime.^18-23^ Furthermore, during the pandemic, schools, social and hospitality venues remained closed. These massive changes have created shifts in exposure to work-life conflict that potentially contributed to well-being.^24^

 Following the discussion so far, we aim to explore the impact of *WFH* on financial and mental well-being. Based on earlier studies, we expect to find a negative impact of *WFH* on mental health, however, this will depend on the frequency of *WFH*. In particular, the mental well-being and job satisfaction are negatively correlated with high levels of *WFH* frequency.^8,25^ However, these studies have mainly employed cross-sectional data, and they explored *WFH* schemes that were planned and known beforehand, while our study explores a sudden and unexpected shift from working at an employer’s premises to WFH. Furthermore, we aim to compare the impact of different intensity levels of *WFH* on well-being, such as working always from home or on occasion.

## Methods

###  Data

 The empirical analysis relied on data derived from the Understanding Society-UKHLS, a nationally representative survey of approximately 30 000 households started in 2009. For the purpose of our identification strategy, we used waves 7-9 for the pre-COVID-19 period, over the years 2015-2019, combined with the Understanding Society-UKHLS COVID-19 survey conducted in April of 2020. The COVID-19 study is an integral part of the UKHLS and it includes all members of the main UKHLS sample who have participated in at least the last two waves of data collection. The design of the UKHLS COVID-19 survey targets to make only minimal adjustments in the field questionnaires to ensure comparability of the data collected in the previous waves. The purpose of the design in the UKHLS COVID-19 survey is to cover the dynamic impact of the pandemic on the welfare of individuals and their families in the United Kingdom.^26^ Overall, the design and the variables remain the same with the UKHLS, hence, researchers can link the data from COVID-19 survey to answers that respondents have given in previous, and also future waves, of the UKHLS.

###  Methodology

 The difference-in-differences (DiD) strategy employed to estimate the effect of WFH on financial and mental well-being was:


(1) $$SWB_{irt}=\beta_{0}+\beta_{1}WFH_{irt}+\beta_{2}covidlock_{irt}+\beta_{3}\left(WFH_{irt}.\;covidlock_{irt}\right)+\beta^{\prime }X_{irt}+\theta_{t}+l_{r}+u_{irt}$$

Where *SWB* denotes the subjective financial or psychological well-being for individual *i* in region *r* and time-wave *t*. For the financial well-being, we created a dummy variable from the question about the individual’s subjective future financial situation. The variable takes a value of 1 if the future financial situation will be worse off and equals 0 if the financial situation will be same or better off. For the psychological well-being, we used the GHQ-12, a well-documented measure of the individual’s psychological and mental well-being, which has been used extensively in various fields, including epidemiological, psychological, social and economic sciences.^27^ GHQ-12 takes values between 0 (excellent well-being) to 12 (very poor well-being).

 Variable *WFH* denotes whether the respondent works from home, and we explore two cases. In the first case, *WFH* takes a value of 1 for those who were never WFH before the lockdown period and work always from home during the COVID-19 period. *WFH* takes a value of 0 for the respondents who never worked from home in both pre-COVID-19 and COVID-19 periods. In the second case, *WFH *takes a value of 1 for those who never work from home before the COVID-19 lockdown period, but they work occasionally from home during the COVID-19 period, while the comparison group remains the same; those who never work from home. Variable *covidlock* takes a value of 1 for the COVID-19 period and 0 for the pre-COVID-19 period. Parameter *β*_3_ is the DiD estimator that identifies the effect on the outcome variables of WFH compared to those who never work from home. The set *l*_r_ indicates the area-government region fixed effects, and time dummies, specifically the month and the year of the interview, are expressed by the set *θ*_t. _The control variables in vector **X** include gender, age, ethnicity, the child in the household, the standard occupational classification code that classifies workers into occupational categories, and the standard industrial classification code, such as agriculture, wholesale, retail, finance, insurance, health and legal services among others.

 We limited the sample of our analysis to those that have non-missing values in all four waves of the survey and were in employment before and after the COVID-19 period. This resulted in 14 520 observations and 3630 individuals when we considered those who work only from home, and 12 144 observations and 3036 individuals when we considered the respondents who work from home on occasion. We clustered the standard errors at the household level and to avoid biased statistical inference and sample attrition we adjusted our regressions accounting for the weight of the survey design.^28^ We estimated regression (1) using the ordinary least squares (OLS) method, and as a robustness check we repeated the estimates using the Fixed Effects OLS (FE-OLS) method.

 One common approach is testing the parallel trends assumption by using leads and lags of the DiD estimator and testing whether there is an anticipatory effect. However, since we have only one post-shock period, -the lockdown period,- we did not implement this test. An alternative way was to use interaction terms of the *WHF* and the time trend variable *t* (see Angrist and Pischke, 2008 for more details) as^29^:


(2) $$SWB_{i,r,t}=\sum_{t=q}^{n}\beta_{q}\left(WFH_{i,r,t-q}.\;t_{t-q}\right)+\beta^{\prime }X_{i,r,t}+\theta_{t}+l_{r}+u_{i,r,t}$$

Where *CS*_i,r,t–q _*∙ t*_t–q_ is showing whether the *WFH*-COVID-19 lockdown is switched on in period *t*, and the lags of the *WFH *are expressed respectively by *q* = 0, 1, 2 where *q* = 0 corresponds to the COVID-19 lockdown period and lags *q* = 1,2 correspond to waves 8-9. We performed a joint hypothesis test for the coefficients of the lagged interaction terms of *WFH* and the time trend *t*, where the null hypothesis implies that the parallel trends assumption holds. We should note that we did not include the interaction term for wave 7, which corresponds to the lag *q* = 3, as this was dropped due to multicollinearity.

 One issue that may pose a threat to the identification strategy is that some workers may have chosen to work from home. In the United Kingdom, the implementation of *WFH* was strongly recommended, while local, national and global companies had rolled-out mandatory work-from-home policies. Furthermore, to reduce endogeneity, we have removed workers who are shielded, as they can choose to work from home, because they are clinically extremely vulnerable and at a high risk from the coronavirus. Another issue is the potential selection bias, where the *WFH* group may include workers belonging to the higher parts of the wage distribution, while the comparison group may consist mainly of healthcare workers and those employed in the retail sector belonging to the lower end of the wage distribution. However, both groups comprise workers belonging to various parts of the wage distribution. In particular, while the group of respondents working at the employer’s premises consists of low-wage workers, there are also respondents employed in managerial positions in the real estate sector and health services. On the other hand, *WFH* includes managers, academics and white-collar professions that belong to the upper levels of the wage distribution. However, low-wage workers such as customer service representatives, administrative and secretarial assistants were also WFH during the COVID-19 pandemic. In particular, almost the 57% of those employed in managerial, professional, and technical occupations were WFH, roughly 52% were employed in administrative and secretarial occupations, followed by almost 31% of those working as customer service representatives, who belong to lower parts of the wage distribution.

 Furthermore, frontline workers, which have been designated as “key workers,” such as doctors, police officers, firefighters and paramedics who belong in the medium and upper levels of the income distribution, were much less likely to work from home. Moreover, the average wage of those WFH and those who do not work from home is respectively 1570 and 1450, while the average wage for those WFH on occasion is 1590. The similarities in wages across the three groups is explained by the fact that almost half of those employed in managerial and higher professional occupations as well as those employed in occupations belonging to the low and middle parts of the wage distributions were WFH. Nevertheless, our aim is to highlight that even though those WFH report a higher positive perception about the future financial situation, they are also those who present higher levels of mental distress.

## Results and Discussion

Table 1 shows the averages and standard deviations of the two outcomes explored in the study for the two cases of the “WFH” and the comparison group described in the previous section. In the first case, we observe that the negative perception about the future financial situation increased from 0.12 to 0.14. The respective increase for the comparison group was 0.22 from 0.10, implying that 22% of the comparison group reports a negative perception about the future financial situation during the COVID-19 period compared to 10% before COVID-19.

**Table 1:** Outcome Variables Means and Standard Deviations

	**First Group: Never Worked From Home in the Pre-COVID-19 Period and Always Work From Home During COVID-19 Period**		**Second Group: Never Worked From Home in the Pre-COVID-19 Period and Work Occasionally From Home During the COVID-19 Period**		**Comparison Group: Never Work From Home in Both Pre-COVID and COVID-19 Periods**	
**Future financial situation (1 for worse off)**	Average	Standard deviation	Average	Standard deviation	Average	Standard deviation
Pre-COVID-19 period (2015-2019)	0.12	0.33	0.11	0.32	0.10	0.30
COVID-19 period	0.14	0.35	0.15	0.37	0.22	0.41
T-test statistic for the first and comparison group in the pre-COVID-19 period	0.9954 [.3344]		T-test statistic for the second and comparison group in the pre-COVID-19 period	1.0150 [.3102]		
T-test statistic for the first and comparison group in the COVID-19 period	-4.4165 [.0000]		T-test statistic for the second and comparison group in the COVID-19 period	-2.7095 [.0068]		
**GHQ-12 caseness**	Average	Standard deviation	Average	Standard deviation	Average	Standard deviation
Pre-COVID-19 period (2015-2019)	1.77	3.03	1.70	2.80	1.64	2.92
COVID-19 period	3.09	3.32	2.77	3.17	2.69	3.23
T-test statistic for the first and comparison groups in the pre- COVID-19 period	1.0801 [.2801]		T-test statistic for the second and comparison groups in the pre- COVID-19 period	0.9374 [.3486]		
T-test statistic for the first and comparison groups in the COVID-19 period	2.9156 [.0035]		T-test statistic for the second and comparison groups in the COVID-19 period	1.2480 [.2121]		

Abbreviations: COVID-19, coronavirus disease 2019; GHQ, 12-item General Health Questionnaire. *P* values within the square brackets.

 On the other hand, we observe a large drop in the well-being of those working always from home, implying that even though the perception on the financial situation remains almost the same, the psychological well-being becomes worse, increasing from 1.77 to 3.09. When we consider the second case we described in the previous section, the perception about the future financial situation is better during the COVID-19 period, compared to those who never work from home, while they do not present any differences in the mental well-being.

 To confirm the differences in the average values among the various groups explored, we have estimated the *t *test for the difference in means. In particular, the *t *test for the difference in means of the future financial situation, between those working always from home and those who never work from home, is 0.9954 and the *P *value is.3344 during the pre-COVID-19 period, and it becomes -4.4165 with a *P *value of zero during the COVID-19 period. Similarly, the *t *test for the mean difference of the future financial situation between those WFH occasionally and the comparison group (never work from home) is 1.0150 (*P *value = .3102) and -2.7095 (*P *value = .0068) respectively in the pre-COVID-19 and COVID-19 period.

 Based on the *t *test, we observe that there are no differences in the average values of GHQ-12 between the first and comparison group (*t *test = 1.0801 and *P *value = .2801) and between the second and the comparison group (*t *test = 0.9374 and *P *value = .3486) during the pre-COVID-19 period. When we consider the average differences between those working always from home and those never work from home, during the COVID-19 period, the *t *test becomes 2.9156 with a *P* value of 0.0035. On the other hand, we find no differences in the average GHQ-12 values between those WFH occasionally and the comparison group, which is confirmed by the value of *t *test, equals 1.2480 (*P *value = .2121).

 In Table 2 and panel A, we report the DiD estimates and observe that those who have changed the working mode to work from home are less likely to report that their financial situation in the future will become worse by almost 10.40 percentage points compared to those who never work from home. This can be due the fact that those individuals are more confident that in the future they will be able to earn and ensure their jobs, even further lockdowns could be implemented. In other words, in the case of lockdowns they probably believe that they have a lower probability of being laid off. Those who never work from home may feel more insecure about their jobs, especially in the case where they cannot shift their working environment from the employer’s premises to their home.

**Table 2:** DiD Estimates

**Panel A: First Group OLS Estimates**		**Panel B: Second Group OLS Estimates**	
**Dependent Variable: Perception of Future Financial Situation **		**Dependent Variable: Perception of Future Financial Situation **	
DiD estimator *β*_3_ (WFH * covidlock)	-0.1043*** (0.0339)	DiD estimator *β*_3_ (WFH * covidlock)	-0.0699** (0.0287)
No. observations	14 520	No. observations	12 144
R-square	0.0294	R-square	0.0322
F-Test for the parallel trends assumption	1.0417 [.3531]	F-Test for the parallel trends assumption	0.8235 [.4409]
**Dependent Variable: GHQ-12 Caseness**		**Dependent Variable: GHQ-12 Caseness**	
DiD estimator *β*_3_ (WFH * covidlock)	0.3625** (0.1432)	DiD estimator *β*_3_ (WFH *covidlock)	0.0804 (0.0529)
No. observations	14 520	No. observations	12 144
R-square	0.0378	R-square	0.0384
F-Test for the parallel trends assumption	0.9690 [.9416]	F-Test for the parallel trends assumption	0.2918 [.7492]
**Panel C: First Group FE-OLS Estimates**		**Panel D: Second Group FE-OLS Estimates**	
**Dependent Variable: Perception of Future Financial Situation **		**Dependent Variable: Perception of Future Financial Situation **	
DiD estimator *β*_3_ (WFH * covidlock)	-0.1065*** (0.0169)	DiD estimator *β*_3_ (WFH * covidlock)	-0.0716** (0.0320)
No. observations	14 520	No. observations	12 144
R-square	0.0118	R-square	0.0133
F-Test for the parallel trends assumption	0.0317 [.7361]	F-Test for the parallel trends assumption	0.6838 [.5083]
**Dependent Variable: GHQ-12 Caseness**		**Dependent Variable: GHQ-12 Caseness**	
DiD estimator *β*_3_ (WFH * covidlock)	0.3726*** (0.1219)	DiD estimator *β*_3_ (WFH * covidlock)	0.0782 (0.0543)
No. observations	14 520	No. observations	12 144
R-square	0.0081	R-square	0.0349
F-Test for the parallel trends assumption	0.0262 [.8234]	F-Test for the parallel trends assumption	0.4922 [.6216]

Abbreviations: WFH, working from home; DiD, difference-in-differences; GHQ, 12-item General Health Questionnaire; OLS, ordinary least squares. Standard errors in the parentheses and clustered at the household level. P-values within the square brackets. *** and ** indicate significance at 1% and 5% level. Regressions are weighted by the sampling survey weight. *Note*: The first group in panels A and C refers to those who never worked from home during the pre-COVID-19 period and they work always from home during the COVID-19 lockdown period. The second group in panels B and D refers to those who never worked from home during the pre-COVID-19 period and they work from home occassionally during the COVID-19 lockdown period.

 However, it is remarkable that we observe a large drop in the mental well-being, expressed by the GHQ-12, by roughly 36 percentage points. While there is no clear explanation and we do not further investigate it in this study, this drop in the mental well-being can be due to social isolation from co-workers, lack of contact with managers that may limit their opportunities for promotion, and stress resulting from additional workload and overtime.^7,22,30-31^ However, this information is unavailable in the data employed in the empirical work, and thus, one of the limitations of this study is that we are unable to identify the possible mechanisms of the drop in mental well-being. Nevertheless, it should be noted that all respondents report a decline in their psychological well-being due to the coronavirus pandemic, but those WFH have experienced a larger drop. In panel B, we report the results when we consider those who work from home occasionally, and we conclude that those who never work from home have more concerns about the future financial situation, however, we find no differences in the GHQ-12 between the two groups. In particular, in the second case, both groups have experienced a similar drop in GHQ-12. To recall, in Table 1 the average GHQ-12 value for those WFH occasionally increased in COVID-19 period from 1.56 to 2.77, while the respective increase for those never worked from home is 2.69 from 1.64, and we have shown that the differences are statistically insignificant.

 In panels C and D of Table 2, we repeat the estimates in panels A and B using FE-OLS as a robustness check, to account for unobserved time-invariant characteristics. Our results remain robust, as the DiD estimators are very close to those found in panels A and B. According to the pre-treatment *F-statistic* tests and the *P *values, we cannot reject the null hypothesis, implying that the parallel trends assumption holds in all cases. In this case, we test the joint significance of the DiD estimated coefficients with 3 and 4 lags, corresponding to the periods 1 and 2 in Figure.

**Figure:**
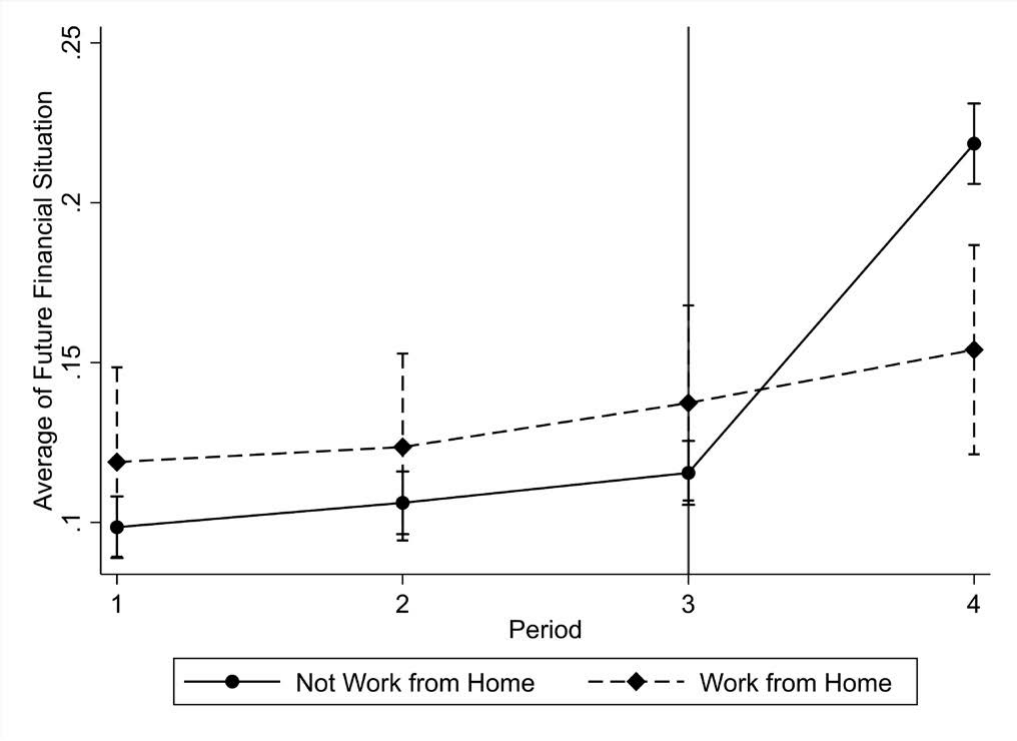
Perception About the Future Financial Situation by Working Always From Home Status.

 While we present only the case of the perception of future financial well-being for those who work from home due to space limitations, corresponding to the panel A of Table 1, we should note that the remained figures confirm the parallel trends assumption test in panels B-D in Table 2 (for more details see Supplementary file 1). The periods 1-3 correspond to waves 7-9 and period 4 refers to the COVID-19 period. Furthermore, we have also estimated the DiD regressions without controls and the DiD estimator remains almost the same, which further supports the robustness of our results and the strength of the research design. Thus, overall, it seems that both groups of workers, those WFH and those who never work from home, would follow the same trend in the two outcomes that we explore in the absence of the lockdown and the implementation of WFH.

## Conclusion

 The findings have important implications for dealing with *WFH* both during the pandemic but also in its aftermath, as *WFH* is most definitely a work arrangement that is now here to stay. The main concluding remark of this study is that those who never work from home are more concerned about their future financial situation compared to those who have experienced a shift from working at the firm’s premises to working always from home. On the other hand, those who work always from home experience lower levels of mental well-being, measured by the GHQ-12. This can be explained by the fact that apart from the potential social isolation and overtime we discussed earlier, those workers were not WFH at all, and suddenly they were moved into an environment they had never before experienced. Although this concluding remark is not directly supported from the results and the data employed in the empirical work, earlier studies may support our initial findings. In particular, these studies suggest that *WFH* has downsides such as the feeling of social and occupational isolation stemming from the fact that employees WFH are away from their managers and colleagues.^32-34^

 However, the results differ when we consider an alternative frequency of *WFH*. In particular, we found no differences in the mental well-being between those WFH on occasion and those working always at the employer’s premises, while those WFH have a better perception about their future well-being during the COVID-19 period. Therefore, the frequency of *WFH* may contribute to well-being as previous studies show that it is important to achieve well-being primarily through structuring employees’ days.^33,34^ It appears that the most successful way for workers to enhance their job performance and well-being, is to structure their days in a way that allows for a better balance between life and work demands. ^20,34,35^ This involves different intensity levels of *WFH*, as we have explored in this study, and in particular, those who work always from home or on occasion.

 Hence, the question is not whether *WFH* is good or bad, as it can have both benefits and undesirable consequences. Instead, organisations should account for the negative effects of *WFH* and implement this employment scheme considering a frequency that does not have detrimental effects on mental health, while maintaining worker’s high levels of job satisfaction and perception about the future financial situation. Even though under the strict guidelines workers are required to work from home wherever possible, the findings of this study may have implications in future implementations of this employment scheme under both normal circumstances, and exceptional shocks, such as the COVID-19 scenario.

## Ethical issues

 An ethical approval was not necessary because we only used secondary data provided from the UK Data Service.

## Competing interests

 Authors declare that they have no competing interests.

## Authors’ contributions

 EG and OO conceptualized the manuscript. EG has acquired the data and prepared the STATA do files. EG and OO prepared the initial manuscript and both authors have read and approved the final manuscript.

## Supplementary files

Supplementary file 1 contains Figures S1-S3.
