# Supplementary material for: Implications of COVID-19: The Effect of Working From Home on Financial and Mental Well-Being in the UK
Source: Int J Health Policy Manag. 2021 Apr 21;11(9):1635–41. doi: 10.34172/ijhpm.2021.33 (PMC9808217; doi:10.34172/ijhpm.2021.33)

Article title: Implications of COVID-19: The Effect of Working From Home on Financial and Mental Well-Being in the UK

Journal name: International Journal of Health Policy and Management (IJHPM)

Authors' information: Eleftherios Giovanis[1]*, Oznur Ozdamar[2]

[1]Department of Public Finance, Nazilli Faculty of Economics and Administrative Sciences, Aydın Adnan Menderes University, Aydın, Turkey.

[2]Department of Economics, Faculty of Economics and Administrative Sciences, Izmir University Bakırcay, Izmir, Turkey.

(*corresponding author: egiovanis@adu.edu.tr)

Supplementary file 1

Figure S1. GHQ-12 Caseness by working always from home

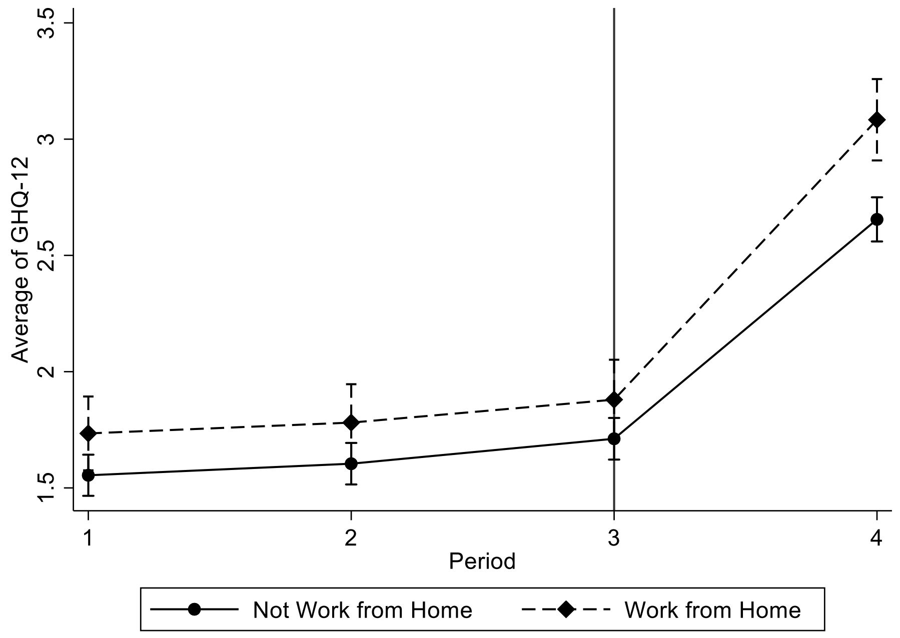

Figure S2. Perception about the future financial situation by working from home on occasion

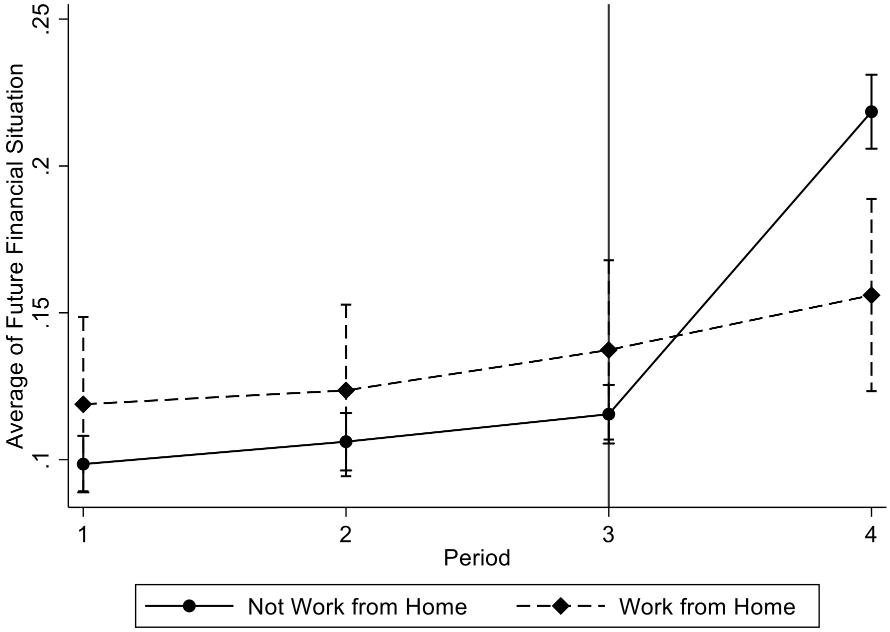

Figure S3. GHQ-12 Caseness by working from home on occasion

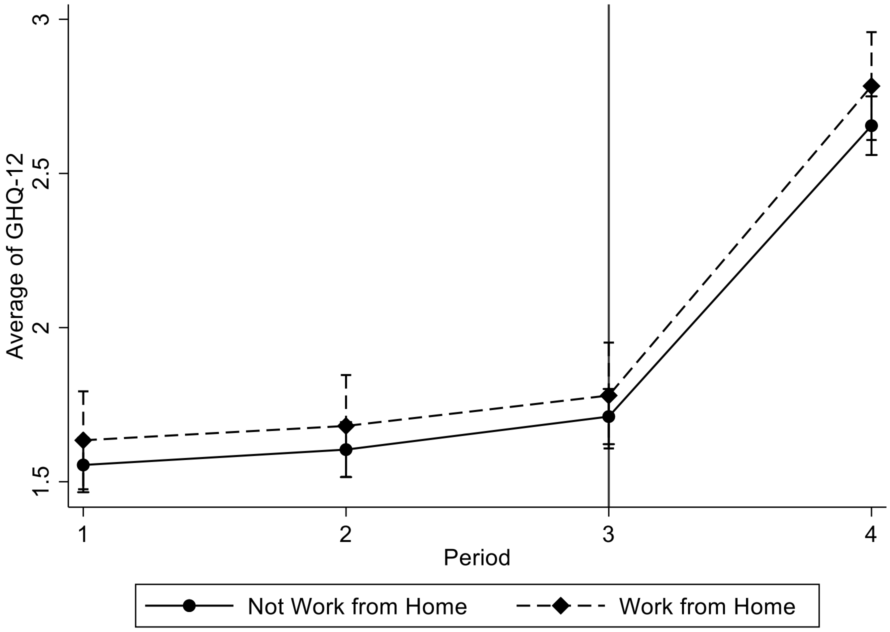

Supplement: Supplementary file 1 — contains Figures S1-S3. [file ijhpm-11-1635-s001.pdf]
